# Electrokinetic Properties of Mesoporous Vitreous Membranes Doped by Silver-Silver Halides

**DOI:** 10.3390/membranes13020126

**Published:** 2023-01-19

**Authors:** Ludmila Ermakova, Anastasiia Kuznetsova, Marina Girsova, Anna Volkova, Tatiana Antropova

**Affiliations:** 1 Institute of Chemistry, Saint Petersburg State University, Saint Petersburg 199034, Russia; 2 Laboratory of the Physical Chemistry of Glass, Grebenshikov Institute of Silicate Chemistry of the Russian Academy of Sciences, Saint Petersburg 199034, Russia

**Keywords:** membrane, porous glass, silver halide, specific electrical conductivity, efficiency coefficient, zeta-potential

## Abstract

Silver/silver halide materials are considered as efficient and highly stable plasmonic photocatalysts for the organic pollutant degradation and hydrogen evolution from water splitting under solar irradiation, and they possess promising antibacterial activity. Ordered mesoporous silica materials including porous glasses are considered as the most promising template for silver-containing structures. In the present work, Ag/AgHal-doped (Hal = Cl, Br) vitreous membranes on a base of the mesoporous glasses were prepared via step-by-step single-stage impregnation procedure. The chemical and phase composition of the modified membranes were identified by the X-ray photoelectron spectroscopy, the X-ray diffraction and the energy-dispersive X-ray spectroscopy. The structure and morphology of inner membrane space were studied by the scanning electron microscopy. Electrokinetic properties of the silver-containing vitreous membranes were determined by the differential method and the streaming potential method. The inner membrane space is modified unevenly with appearance of the clearly defined regions with different silver content. The formation of the Ag/AgCl clusters along with the individual nanoparticles over thickness of the 1-mm membrane with mean pore radius of 23 nm was detected. The modification of the pore space by Ag-containing structures and the type of halogen ion almost do not affect the electrochemical behavior of the mesoporous vitreous membranes.

## 1. Introduction

As known, silver/silver halide (Hal = Cl, Br, I) nanoparticles (NPs), due to the surface plasmon resonance (SPR) effect, exhibit excellent photoactivity [1,2,3]. Such silver-containing materials are considered as efficient and highly stable plasmonic photocatalysts for the degradation of organic pollutants and hydrogen evolution from water splitting under solar irradiation [4,5,6,7,8,9,10]. Ag/AgHal nanoparticles also possess promising antibacterial activity [11,12,13]. However, NPs tend to agglomerate in liquid media, which negatively affects the photoactivity. Therefore, up to now, numerous works have been devoted to enhancing the photocatalytic activity by loading the Ag/AgHal nanoparticles on solid supports [14,15,16], especially porous ones. 

Ordered mesoporous silica nanoparticles (SBA-15, MCM-41 etc.) are considered as the most promising template for silver/silver halide NPs owing to the large surface area, high chemical and thermal stability, uniform pore size, low cost and good biocompatibility [9,17]. In turn, the incorporation of Ag-containing nanoparticles in pore space of optically transparent SiO_2_ in comparison with the core-shell structures is beneficial for SPR effects owing to better particle distribution homogeneity, photo- and chemical stability, stronger binding between Ag/AgHal and the substrate, and for antibacterial activity due to slower silver ion release rate.

Thus, Ag/AgHal/SiO_2_ composites, due to the remarkable adsorption properties and stable photocatalytic activity under visible light, can be successfully used in water purification for the degradation of organic pollutants such as dyes [18,19], phenols [20], in deep desulfurization processes [21], and in various antibacterial applications [22]. Despite the extensive research in a field of the synthesis and the study of the functional properties of these composite particles, there are noticeably fewer publications devoted to membrane systems containing Ag/AgHal. For preparation of the multifunctional composite membranes for concurrent water filtration, photocatalytic pollutant degradation under visible light and disinfection in a sustainable way, both organic and inorganic templates are used [23,24,25,26]. However, there is a lack of information about massive porous silica templates. 

Among the porous silica materials with the confined geometry of the pore space, porous glasses (PGs), the products of the acid leaching of the two-phase alkali borosilicate glasses [27] have promising perspectives. In particular, due to the opportunity to produce the PGs not only in the form of powders (particles) but also of the massive products of the various geometric shapes (disks, plates and so on), porous glasses are successfully used as vitreous membranes [28,29,30]. 

We demonstrated in our earlier works [31,32,33] that porous and quarzoid-like composites, containing silver halides and metallic silver or silver oxide as a product of partial decomposition of the AgHal owing to photolysis or thermolysis, can be successfully prepared via step-by-step single-stage impregnation of the porous glass discs or plates with silver nitrate and potassium halides followed by heat treatment. The electrokinetic characteristics of the mesoporous vitreous membranes doped by silver iodide were studied in our work [34]. In this regard, this was of interest to identify the influence of the halide ion on the regularities of the mesoporous vitreous membrane modification and on their electrokinetic properties.

In the present work we obtained the Ag/AgHal-doped (Hal = Cl, Br) mesoporous silica membranes and, for the first time, studied their electrochemical characteristics in electrolyte solutions (NaNO_3_, AgNO_3_) along with the morphology of the pore space, chemical and phase silver-modified membrane composition, and compared them with those for the membranes modified by silver iodide. We believe that such membranes may have potential applications in the medicine and environmental processing fields.

## 2. Materials and Methods

### 2.1. Materials

AgNO_3_, KCl, KBr, NaNO_3_ solutions were prepared using special pure grade reagents and deionized water (the UVOI-“M-F” installation, water conductivity is less of 1.5 × 10^−6^ Ohm^−1^cm^−1^).

### 2.2. Preparation of Silver-Containing Vitreous Membranes 

The porous glasses (PGs) in the form of discs of 30 mm in diameter and about 1 mm thickness were used as base membranes. So-called microporous (MIP) glasses (according S.P. Zhdanov terminology [35]) were obtained according to the standard procedure [36,37,38] from the monolithic two-phase alkaline borosilicate glasses with a two-frame structure of 8V-NT composition (wt %): 6.74 Na_2_O—20.52 B_2_O_3_—0.15 Al_2_O_3_—72.59 SiO_2_ [36] through chemically etching (leaching) in 3 M HNO_3_ aqueous solutions at boiling. Next, the leached samples were washed with distilled water and dried in an air drying box at 120 °C for 1 h [39]. As a result, porous MIP glasses [37,40] were obtained.

Macroporous (MAP) glasses were prepared from MIP ones by additional treatment in 0.5 M KOH solution at 20 °C, with subsequent washing in distilled water and air drying at 120 °C for 1 h. Secondary silica is removed from the pore space [37] of the MIP glasses during their alkali treatment as a result the average pore size increases [37,38]. We marked our membranes as MIP and MAP according to their preparation technique.

Silver-containing vitreous membranes were prepared by step-by-step impregnation with aqueous solutions of 0.6 M AgNO_3_ and 0.6 M potassium halide (KCl or KBr) solutions at 50 °C under thermostatic conditions without forced stirring for 20–45 min, according to the stoichiometry of the chemical reaction:

AgNO_3_ + KHal = AgHal↓ + KNO_3_ (Hal = Cl, Br)

followed by drying at 120 °C for 1 h by analogy with the procedure [39,41].

### 2.3. Characterization

The XPS and XRD measurements were carried out for the powders of as-prepared silver-containing grinded membranes. For this, the pieces of the modified membranes were thoroughly grinded in an agate mortar in a darkened room to avoid the light exposure. The agate mortar was in addition covered with a black dense film.

The crystalline phase structure was determined by X-ray diffraction (XRD) on the Desktop powder diffractometer Bruker D2 Phaser (Bruker AXS, Karlsruhe, Germany) using CuK_α_ radiation in the 2 theta range of 5–85° in the Centre for X-ray Diffraction Studies at the Research Park of Saint Petersburg State University (SPSU). Phase identification was performed using Powder Diffraction File (PDF-2, 2020): potassium bromide No.01-071-3752 and 00-004-0531, sylvite syn. No. 00-041-1476, bromargyrite syn No. 00-006-0438 and 01-071-3754, chlorargyrite syn No. 00-031-1238, silver-3C syn. No 00-004-0783, 03-065-2871 and 01-071-3762. The surface electronic state was identified through X-ray photoelectron spectroscopy (XPS) performed on an integrated photoelectronic and scanning Auger electron spectrometer Thermo Fisher Scientific Escalab 250Xi (Thermo Fisher Scientific, Cambridge, United Kingdom) using Al Kα radiation as the excitation source in the Centre for Physical Methods of Surface Investigation, SPSU.

The structure and morphology and the element analysis of the Ag-modified membranes were studied by scanning electron microscopy (SEM) and the energy-dispersive X-ray spectroscopy (EDX), respectively, using a Carl Zeiss Merlin field-emission scanning electron microscope (Carl Zeiss Merlin, Oberkochen, Germany) equipped with INCAx-act spectrometer with an Analytical Silicon Drift Detector (SDD) INCAx-ac (Oxford Instruments INCAx-act, Oxford, United Kingdom) in the Interdisciplinary Resource Centre for Nanotechnology, SPBU. 

The electrokinetic properties of Ag-modified vitreous membranes were determined both in indifferent electrolyte NaNO_3_ not containing potential determining ions and in AgNO_3_ solutions containing potential determining ions of Ag^+^ at the neutral pH (5.5–5.7) and 10^−4^–10^−1^ M electrolyte solutions. The pH was measured using Seven S-80K Mettler Toledo pH-meter (Mettler-Toledo GmbH, Schwerzenbach, Switzerland). 

The specific electrical conductivity of the membranes κ_M_ was determined by the differential method [28,29] at 20 °C (LOIP—LT-111 thermostat). The measurements of the solution and membrane conductivities were carried out using an immittance meter E7-21 at a frequency 1000 Hz. The error of κ_M_ value did not exceed ± (2–3)%. 

The specific electrical conductivities of the membranes in 10^−4^–10^−2^ M electrolyte solutions were used for the efficiency coefficients α calculation by the Equation (1) [28]:

κ = ακ_V_ = κ_M_β, (1)

where κ and κ_V_ are the specific electrical conductivities of the pore and bulk solutions, respectively, and β is the structural resistance coefficient in 0.1 M electrolyte solution at α = 1.

The electrokinetic potential ζ of the membranes was determined by the streaming potential method (Ag/AgCl electrodes, a Fluke 8846A/Su multimeter) at pressure equal 0.1–2 atm. The error in the streaming potential *E*_S_ values did not exceed 3–5 % for macroporous membranes. For microporous ones the measurement error increased up to 10–15% only in concentrated solutions. However, it has almost no effect on the regularities of the zeta-potential via electrolyte concentration. 

Zeta-potentials were calculated taking into account the surface conductivity and the electric double layer (EDL) overlap using Equation (2) [42]:

ζ = (ηκ*E*_S_/εε_0_*P*)/f(*kr*,ζ,β*), (2)

where η is the fluid viscosity, ε and ε_0_ are the dielectric permeabilities of the medium and vacuum, respectively, *r* is a pore radius, $f(kr{,\zeta ,\beta }^{*})$ is a function that takes into account the effect of the EDL overlap on the measured value of the streaming potential [43], β* = ε^2^ε_0_^2^*R*^2^*T*^2^*k*^2^/F^2^κ_V_η is a parameter taking into account the properties of the electrolyte, and *k* is the Debye parameter. 

## 3. Results and Discussion

### 3.1. Chemical Composition and Structural Parameters 

**Figure 1:**
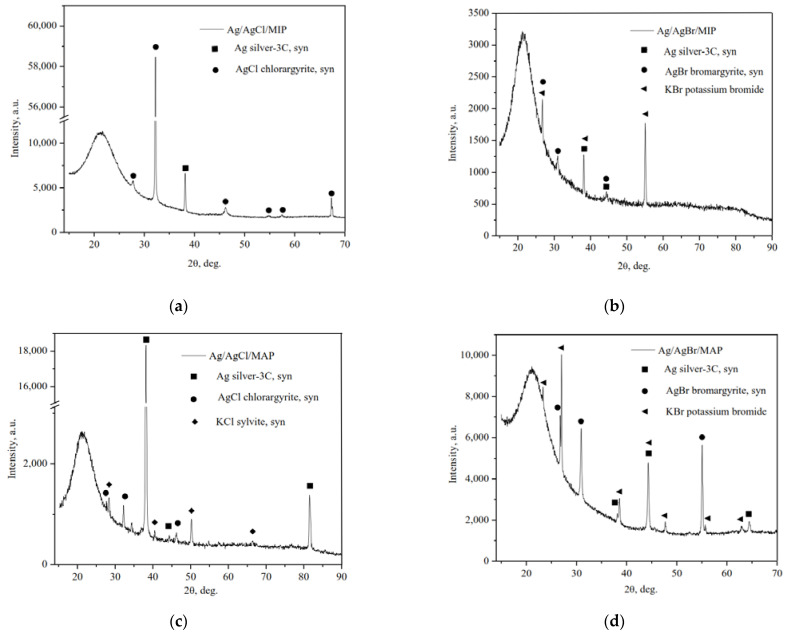
XRD patterns of the as-prepared Ag/AgHal-modified vitreous membranes: Ag/AgCl/MIP (**a**); Ag/AgBr/MIP (**b**); Ag/AgCl/MAP (**c**); Ag/AgBr/MAP (**d**).

The X-ray powder diffraction patterns of the as-prepared Ag-modified MIP and MAP membranes presented in Figure 1 are characterized by a broad peak centered at around 2θ = 23°, which corresponds to amorphous silica SiO_2_. Despite the fact that SiO_2_ is non-crystalline there is the broad peak due to the short-range order. 

As we can see, the typical cubic phase of metallic Ag^0^, space group Fm-3m (225), and the cubic phases of AgCl and AgBr, space group Fm-3m (225), were formed. It should also be noted that the crystalline cubic phases of potassium halides (KCl and KBr, space group Fm-3m (225)) as not reacted component of the final stage of impregnation are also on the diffraction patterns. Thus, it can be seen that as a result of partial photolysis of silver halides a part of ionic Ag^+^ were reduced to metallic silver and Ag/AgHal were formed via simple impregnation procedure, which corresponds with data of the selected area electron diffraction (SAED) for similar materials reported in [31].

**Figure 2:**
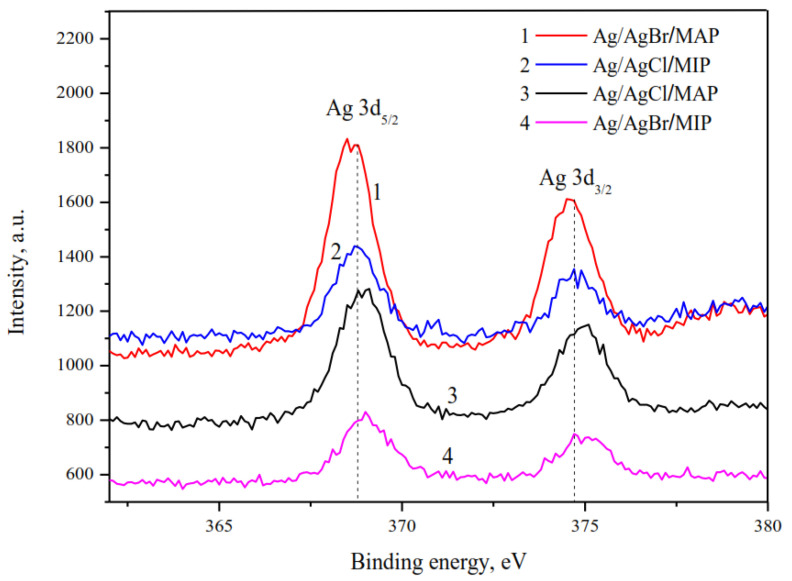
High-resolution XPS spectra of Ag 3d.

XPS analysis of the silver-doped membranes justified silver in metallic form only (Figure 2). The peaks of Ag 3d photoelectron spectrum with binding energy of 368.6–369.0 eV and 374.6–375.0 eV are assigned to Ag 3d_5/2_ and Ag 3d_3/2_ of Ag^0^, respectively [44,45,46]. The ionic form was not detected on the particle surface of grinded membranes in comparison with the entire volume of the sample analyzed by XRD owing to the surface photolysis of silver halides.

**Figure 3:**
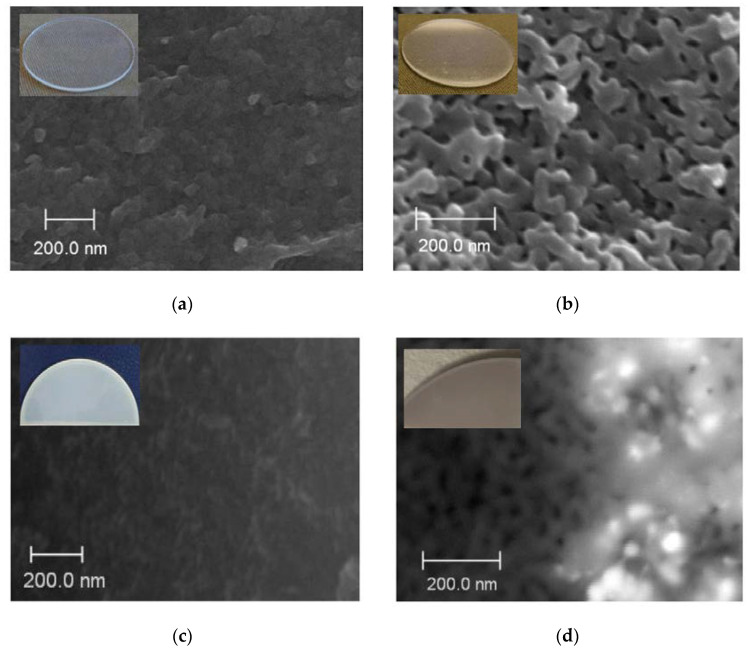
SEM images of the cross-section of the base MIP (**a**) and MAP (**b**) membranes and doped by Ag/AgCl, respectively (**c**,**d**).

SEM images of the cross-section of base MIP and MAP membranes are presented in Figure 3a,b. As seen, for the vitreous membranes a typical two-frame structure is observed that is in good agreement with the data for similar PGs [30,34,42]. It is worth noticing that the mean pore radius *r* of the Ag/AgHal/MIP membranes calculated from the specific surface area within the cylindrical pore model [29,30] is 1.6 nm. For Ag/AgHal/MAP membranes, the *r* values found by liquid filtration [29,30] are 23 nm. The volume porosities determined by the weight method [28] are 0.21–0.22 and 0.56–0.57 for doped micro- and macroporous glasses, respectively. These structure parameters coincide with those for the base membranes (data are presented in [34]) within an experimental error. Therefore, despite the modification of the membrane, their structure parameters such as the mean pore radius and the volume porosity barely change and are in good agreement with SEM data (Figure 3c,d).

**Figure 4:**
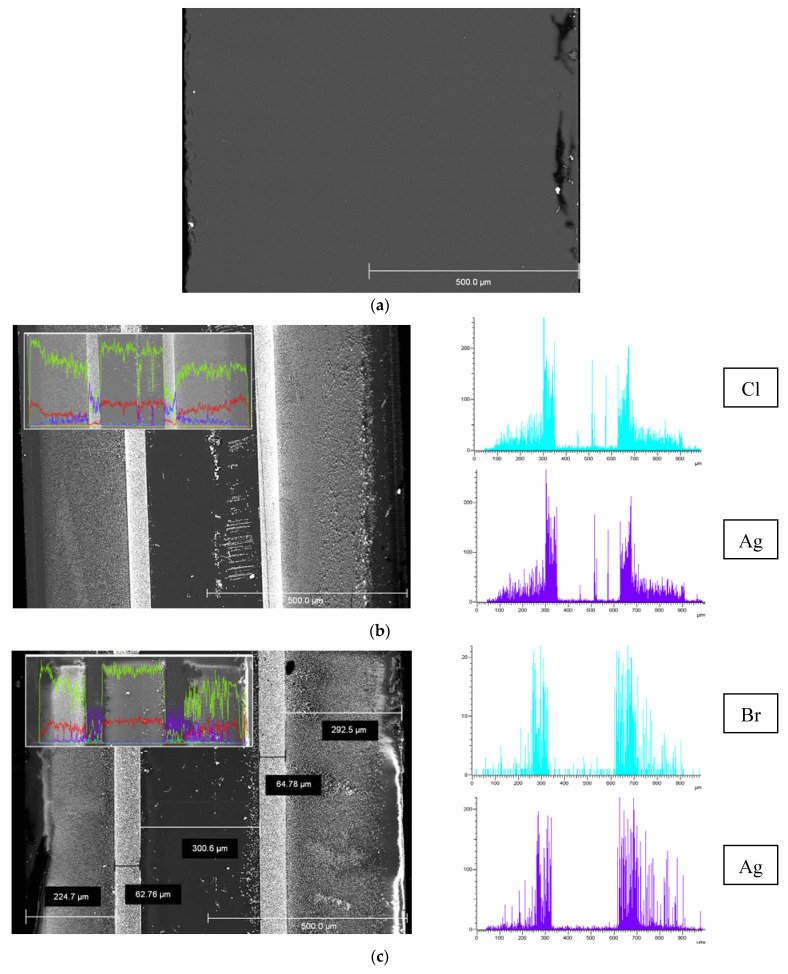
SEM images of the cross-section of the base MIP membrane (**a**) and MIP membranes modified by Ag/AgCl (**b**), Ag/AgBr (**c**) and element distribution over 1 mm thickness (Si—yellow, O—red, Cl/Br—blue, Ag—purple).

SEM images of the cross-section of the base and the composite MIP membranes and EDX distribution of Cl/Br and Ag elements over their thickness are presented in Figure 4. It is seen (Figure 4b,c) that regardless of the halide ion type their distribution profiles over the membrane thickness are similar and coincide with silver distribution. As also seen, there are zones with thickness of about 50–80 μm with a notable high content of dopant distant 250–300 μm from the edge of the composite 1 mm-membranes (Figure 4b,c), in contrast to the uniform structure of the base one (Figure 4a). In turn, there is a negligible content of the modifier in the membrane middle area with thickness of about 300 μm. Thus, doping of MIP membranes containing secondary silica in pore space by the proposed impregnation method results in uneven distribution of silver and halogen elements over the membrane thickness and appearance of the pronounced zones with greatly different Cl/Br and Ag content. 

**Figure 5:**
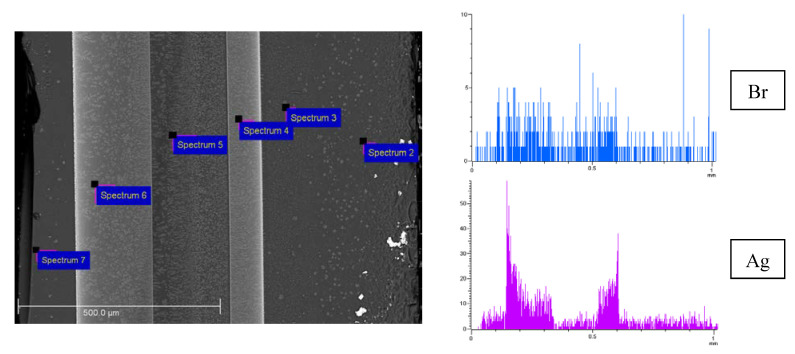
SEM image of the cross-section of the Ag/AgBr/MAP membrane and element distribution over 1 mm thickness (Br—blue, Ag—purple).

**Figure 6:**
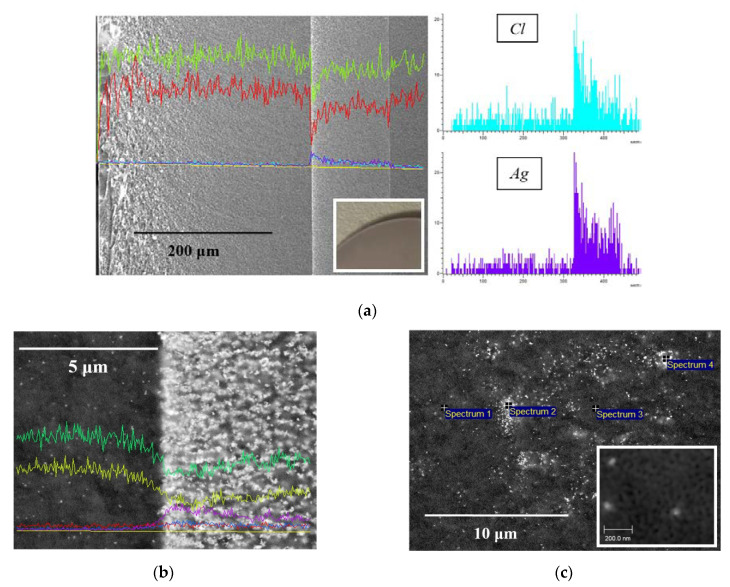
SEM image and element distribution of the cross-section of the Ag/AgCl/MAP membrane: Si—green, O—red, Cl—blue, Ag—purple (**a**), Si—green, O—yellow, Cl—blue, Ag—purple, C—red (**b**)**;** spectra containing (1, 2, 4) and not-containing (3) silver element (**c**).

SEM images of the cross-section of the Ag/AgBr/MAP membrane, Br and Ag element distributions over thickness are presented in Figure 5. As in the case of MIP membranes, a zoned silver distribution is observed. Table 1 shows the EDX results for the surface areas pointed out in Figure 5 (Si and O content are omitted). As seen from spectra 3 and 5, the amount of potassium and chlorine are close, and the ratio of elements is almost 1:1. Silver is determined in spectra 4 and 6. The content of bromine increases with respect to the amount of potassium by the silver content or silver is in excess. In turn, only silver is detected in spectrum 7. Thus, this allows making assumptions about the formation of both silver bromide phase and metallic silver, which is consistent with XRD and XPS. Note that Ag content calculated from EDX data over membrane cross-section is in a range about 0.7–8.2 wt %. Similar regularities were observed for the Ag/AgCl/MAP membrane (Figure 6a).

As seen from Figure 6b,c, SEM images of the Ag/AgCl/MAP cross-section demonstrate mainly the agglomerates of Ag/AgCl nanoparticles unevenly distributed over the membrane thickness. However, the individual nanoparticles (Figure 6b,c) with average size about 30 nm are also observed. It is obvious that the results of EDX and SEM analysis are consistent with each other. Indeed, the number of particles grows dramatically from a near-surface zone with a small content of silver element to a zone with a maximum content. It is worth noticing that EDX data confirm modification of the pore space of the MAP membranes accompanied by a change in their visual transparence (Figure 3b, Figure 5 and Figure 6a).

Thus, the internal membrane space is modified by the proposed impregnation method unevenly with the appearance of zones with different Ag/AgHal content, which is in good agreement with data reported in [47]. This may be due to the precipitation of the silver halide as a result of the reaction between the silver nitrate with the halide solution (KHal) in the pores. Therefore, the pores can be partially “locked”, which makes it difficult for KHal solution to diffuse deeper into the middle of the membrane and react with AgNO_3_. It is possible that for MIP membranes the precipitation is facilitated by the presence in these zones of the layered deposits of the secondary silica (the so-called strata) [48]. The formation of silver-containing compounds mainly at some distance from the membrane edge is most likely due to the washing out of the reagents from the near-surface layer of the porous glass. At the same time, an influence of the so-called “edge effect” for MIP membranes cannot be excluded. The structure of the pore space of MIP glasses close to their outer surface differs from the bulk one; in particular, there is no secondary silica in it. It is likely that the absence of the secondary silica in the pores of MAP membranes is associated with the observed less-ordered distribution of the silver and halogen elements over the thickness (Figure 5 and Figure 6a).

### 3.2. Electrokinetic Characteristics

**Figure 7:**
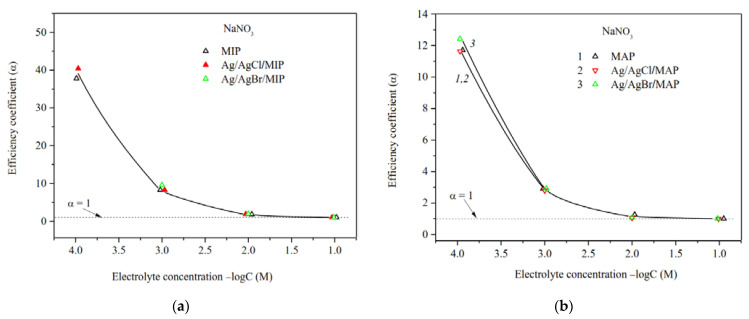
Efficiency coefficients of the base (Ermakova, 2022. Ref. 34) and Ag/AgHal-modified MIP (**a**) and MAP (**b**) membranes vs. NaNO_3_ solution concentration.

**Figure 8:**
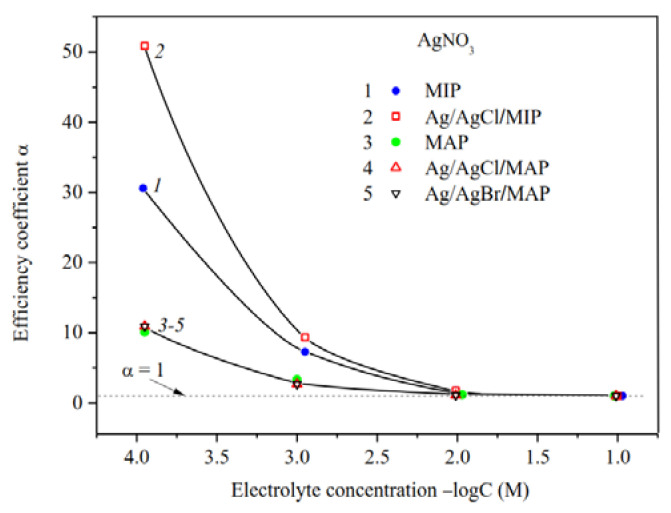
Efficiency coefficients of the base (Ermakova, 2022. Ref. 34), Ag/AgCl/MIP and Ag/AgHal/MAP membranes vs. AgNO_3_ solution concentration.

**Figure 9:**
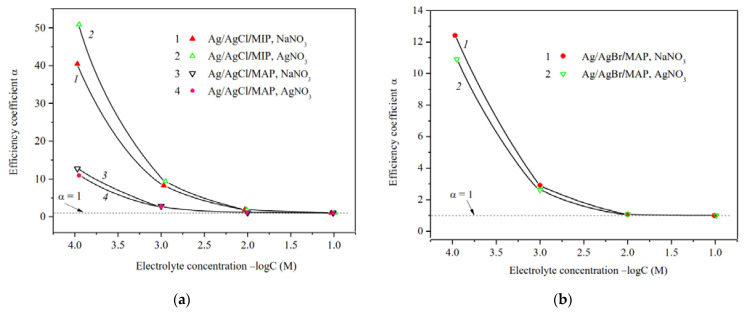
Efficiency coefficients of Ag/AgCl-doped MIP and MAP membranes (**a**) and Ag/AgBr/MAP (**b**) membranes vs. NaNO_3_ and AgNO_3_ solution concentration.

The efficiency coefficients α of Ag/AgHal-containing membranes as well as the dependencies α—log *C* for the base porous matrices reported in [34] are presented in Figure 7, Figure 8 and Figure 9. As seen, the efficiency coefficient values for all vitreous membranes decrease with an increase in the concentration of salt solutions as well as in the pore size in accordance with the classical theories about the contribution of electric double layer (EDL) ions to the electrical conductivity of the pore solution.

The analysis of the concentration dependences of the efficiency coefficient in indifferent electrolyte NaNO_3_ showed (Figure 7a,b) that α values for the base MIP and MAP membranes and Ag/AgHal-doped ones (including Ag/AgI-modified mesoporous membranes, see [34]) almost coincide within the experimental error. In this regard, in a case of the MIP composites in which the Ag/AgHal content is less compared to MAP ones, we have studied further only electrokinetic properties of the Ag/AgCl/MIP membrane. 

Measurements of the specific surface conductivity of membranes in silver nitrate solutions (Figure 8) showed that in a case of the microporous systems (see curves 1, 2) the α values are significantly higher for the Ag/AgCl/MIP than for undoped one at *C* < 10^−2^ M. This indicates an increase in the mobility of EDL ions in the pore space of the doped sample compared to the base vitreous membrane. As seen, for macroporous membranes the efficiency coefficients coincide within the error of the experiment. This means that the electrokinetic properties of the MAP membranes depend mainly on the counterion type rather than the presence (or absence) of dopant. It is worth noticing that the efficiency coefficients for the AgI-doped membranes [34] coincide with ones doped by AgCl and AgBr within the experimental error in the electrolyte containing the potential determining ions of Ag^+^, as well as in the indifferent one.

The predominant influence of the potential determining Ag^+^ ions on the material properties in the electrolyte solution was also found for the Ag/AgCl/MIP membrane. As seen from Figure 9, the efficiency coefficients in diluted NaNO_3_ solutions are less than in AgNO_3_ solutions. This seems to be due to the different structure of secondary silica as well-known to be contained in the pore space [40,49], which depends on the counterion type.

The ratio of the efficiency coefficients for Ag/AgHal-doped macroporous membranes not containing secondary silica remains the same as for the base MAP glasses [34] in sodium and silver nitrate solutions. Thus, α values decrease in diluted AgNO_3_ solutions in comparison with indifferent electrolyte NaNO_3_. The reason for this is probably the slightly higher specificity of the silver ions to the silicate surface in comparison with Na^+^, which results to a decrease in Ag^+^ mobility in the pore space.

**Figure 10:**
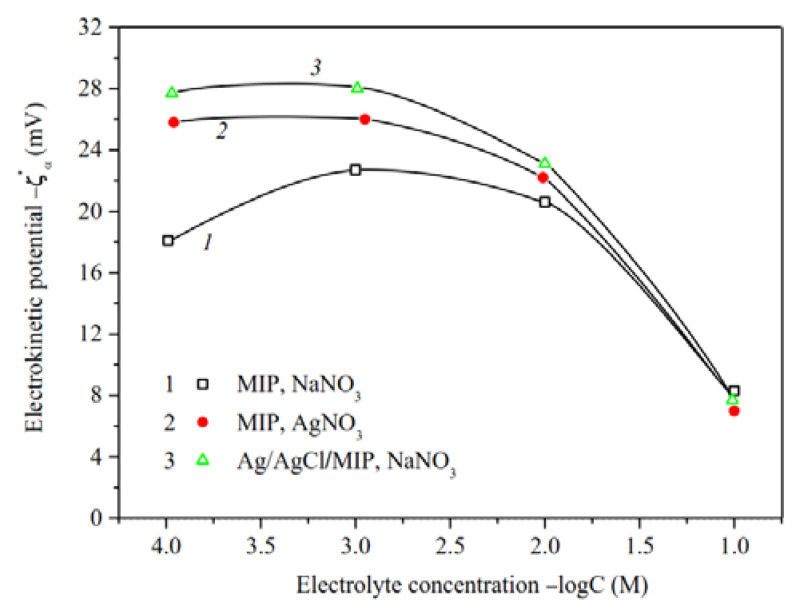
Electrokinetic potential of the base (Ermakova, 2022. Ref. 34) and Ag/AgCl-modified MIP membranes vs. the concentration of NaNO_3_ and AgNO_3_ solutions.

**Figure 11:**
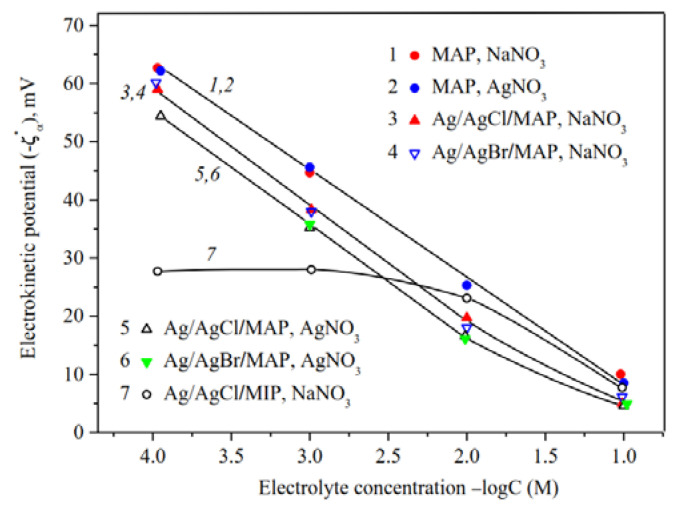
Electrokinetic potential of the base (Ermakova, 2022. Ref. 34), Ag/AgHal/MAP and Ag/AgCl/MIP membranes vs. the concentration of NaNO_3_ and AgNO_3_ solutions.

The electrokinetic potential dependencies on concentration of salt solution for the base [34] and Ag/AgHal-doped membranes are presented in Figure 10 and Figure 11. As seen, ζ-potential is negative for all membranes at the neutral pH regardless of electrolyte composition.

For microporous membranes (Figure 10), absolute values of zeta-potential increase at solution concentration 10^−3^–10^−1^ M, whereas at *C* < 10^−3^ M either decrease or are constant. The observed regularities can be associated both with the shortcomings of the theory of electrokinetic phenomena for small electrokinetic radii *kr* and with the changes in the morphology of the pore space of MIP membranes containing significant amounts of secondary silica [37,40]. The swelling degree of the secondary silica increases with dilution to the greater degree, the more counterion is hydrated (in our case, the sodium counterion), which leads to a displacement of the slipping plane further from the pore channel surface and, therefore, to a decrease in |ζ| values. Apparently this was a main reason of the observed ratio of |ζ|-potentials for the base MIP membranes in NaNO_3_ and AgNO_3_ solutions at *C* < 0.03 M [34]. As seen from Figure 10 (curves 1, 3), modification of microporous membrane with Ag/AgCl results in an increase in absolute zeta-potential at *C* < 10^−^^1^ M in NaNO_3_ solution. It seems to be due to the approaching of the slipping plane to the pore channel surface because of the removal of part of the secondary silica from the pores during treatment of the MIP vitreous membranes with concentrated salt solutions.

As seen from Figure 11, the concentration dependencies of zeta-potential are linear over the chosen electrolyte concentration range for the base macroporous matrices and at *C* < 10^−2^ M for Ag/AgHal/MAP membranes, which coincides with the theoretical concepts about the EDL structure at the interface of the oxide material and electrolyte solution.

The comparison of ζ—log *C* dependences for the base MAP membranes [34] showed that |ζ|—potential are almost the same in NaNO_3_ and AgNO_3_ solutions and coincide with those presented earlier for similar porous glasses in NaCl solutions [50]. Thus, it was found to have negligible effect of silver ion specificity on the electrokinetic potential of the pore surface of MAP membranes, which is characterized by the absence of secondary silica in the pore space.

As seen, the chemical composition of dopant almost has no effect on the absolute value of zeta-potential for Ag/AgHal/MAP membranes regardless of the chosen electrolyte solution, and their |ζ|—potentials decrease in comparison with the base MAP matrices. In turn, the decrease in absolute electrokinetic potential of Ag/AgHal-doped membranes in silver nitrate solution compared to the indifferent electrolyte seems to be caused by the presence of positively charged in AgNO_3_ solution AgHal nanoclusters located in the glass pores. Thus, the electrokinetic potential of macroporous glasses is determined by the absence or presence in the membrane pore space of the silver-containing dopant AgHal. 

Thus, the analysis of the presented data for Ag/AgCl- and Ag/AgBr-doped membranes and comparison of them with the results for Ag/AgI-modified ones (see in [34]) indicate that the type of halogen ion does not significantly affect the electrokinetic behavior of the doped mesoporous vitreous membranes.

As shown in Figure 11, in indifferent electrolyte solution (*C* > 0.025 M) the absolute zeta-potentials are greater for Ag/AgCl/MIP (curve 7) than for Ag/AgHal/MAP membranes. The observed ratio confirms the assumption that doping of MIP glass, in particular, affects the amount and structure of secondary silica in the pore space. In turn, this then reflects in the EDL structure through the change in the electrokinetic potential. For MAP membranes, doping directly affects the characteristics of the pore solid surface.

## 4. Conclusions

The vitreous mesoporous membranes (MIP membranes containing the secondary silica in the pore space and MAP ones without it according to the terminology in [35]) doped by AgCl and AgBr were synthesized via a simple step-by-step single-stage impregnation procedure. The partial photolysis of the silver halides results in the reduction in a part of ionic Ag^+^ to metallic silver and the Ag/AgHal formation. The inner MIP and MAP membrane pore space is modified unevenly with the appearance of regions with different silver content. For MAP membranes, the formation of the Ag/AgCl agglomerates along with the individual nanoparticles with average size about 30 nm are observed. 

The electrokinetic characteristics of the doped MIP as well as the base membranes are determined only by presence and the structure of the secondary silica in the membrane pore space, which depend on the counterion type and the contact time with the concentrated electrolyte solution (for example, during impregnation procedure). In a case of the macroporous membranes, the doping almost does not affect the specific electrical conductivity of the membranes and leads to a decrease in the absolute value of the negative electrokinetic potential, especially noticeable in AgNO_3_ solution containing potential-determining Ag^+^ ions, which also indicates the formation of silver-containing phases within the pores. In turn, the type of halogen ion (Cl, Br and I [34]) does not almost affect the electrokinetic behavior of the doped vitreous membranes. 

Thus, the impregnation procedure used can also be used for the synthesis of the mesoporous vitreous membranes with the electrochemical characteristics close to or slightly different from the base ones; however, containing plasmonic photoactive component. We believe that such membranes may have potential application as bio- and photocatalytic active membranes in the medicine and environmental processing field.

## Figures and Tables

**Table 1:** EDX cross-section element (except for Si, O) atomic concentration (at %) of the Ag/AgBr/MAP membrane.

Spectrum	Element, at %		
	K	Br	Ag
2	0.27	-	-
3	0.42	0.46	-
4	0.63	2.16	1.70
5	1.44	1.29	-
6	0.66	1.91	1.74
7	-	-	0.13

## Data Availability

Not applicable.
